# Amniotic Epithelial Cells Accelerate Diabetic Wound Healing by Modulating Inflammation and Promoting Neovascularization

**DOI:** 10.1155/2018/1082076

**Published:** 2018-08-19

**Authors:** Yongjun Zheng, Shiqing Zheng, Xiaoming Fan, Li Li, Yongqiang Xiao, Pengfei Luo, Yingying Liu, Li Wang, Zhenci Cui, Fei He, Yuhuan Liu, Shichu Xiao, Zhaofan Xia

**Affiliations:** ^1^Burns Center, Changhai Hospital, Second Military Medical University, Shanghai, China; ^2^No. 73051 Troop of the Chinese People's Liberation Army, Xiamen, China; ^3^Department of Obstetrics and Gynecology, Changhai Hospital, Second Military Medical University, Shanghai, China

## Abstract

Human amniotic epithelial cells (hAECs) are nontumorigenic, highly abundant, and low immunogenic and possess multipotent differentiation ability, which make them become ideal alternative stem cell source for regenerative medicine. Previous studies have demonstrated the therapeutic potential of hAECs in many tissue repairs. However, the therapeutic effect of hAECs on diabetic wound healing is still unknown. In this study, we injected hAECs intradermally around the full-thickness excisional skin wounds of db/db mice and found that hAECs significantly accelerated diabetic wound healing and granulation tissue formation. To explore the underlying mechanisms, we measured inflammation and neovascularization in diabetic wounds. hAECs could modulate macrophage phenotype toward M2 macrophage, promote switch from proinflammatory status to prohealing status of wounds, and increase capillary density in diabetic wounds. Furthermore, we found that the hAEC-conditioned medium promoted macrophage polarization toward M2 phenotype and facilitated migration, proliferation, and tube formation of endothelial cells through in vitro experiments. Taken together, we first reported that hAECs could promote diabetic wound healing, at least partially, through paracrine effects to regulate inflammation and promote neovascularization.

## 1. Introduction

Diabetic ulcers are a severe, resistant complication of diabetes mellitus (DM), and about one fourth of DM patients endure diabetic lower extremity ulcers during their whole lives [1]. Diabetic wounds tend to heal slowly and be frequently recurrent, not only leading to increasing cost of medical care but also seriously impairing the quality of life in diabetic patients. Current standard treatments in clinics such as debridement, moist dressings, infection control, or wound offloading have not achieved the satisfactory results. Therefore, we urgently need more effective therapeutic approaches to promote diabetic wound healing.

Stem cells have shown great potential in repairing damaged tissue. Mesenchymal stem cell (MSC) is one of the most widely studied stem cells for wound healing, and several studies have demonstrated inspiring preclinical results through animal models [2–5]. Nonetheless, several arguments still exist against the widely use of MSC in clinics, including the probability of tumorigenesis and low cell production from MSC harvesting. Human amniotic epithelial cells (hAECs) are derived from the amniotic membrane and demonstrate a potential source of stem cells. These cells are nontumorigenic and low immunogenic and possess multipotent differentiation ability [6–8]. Furthermore, hAECs are highly plentiful, and about 1.5 × 10^8^ cells could be separated from each amniotic membrane, which is plenty for using hAECs directly in clinics without amplification in vitro [9]. In this regard, the use of hAECs for regenerative medicine holds great promise.

Recently, the beneficial effects of hAECs in many tissue repairs are reported [10–13]. Although studies have shown that hAECs play their beneficial roles through preventing inflammatory responses, the exact mechanisms of hAECs for promoting tissue repairs remain unclear [11, 14]. A common feature of refractory healing wound is persistent inflammation, impaired vascularization, and decreased expression of growth factors [15]. Whether hAECs play a role in diabetic wound healing associated with inflammation and neovascularization is still unknown.

In this study, we determined whether hAEC transplantation could promote diabetic wound healing by injecting hAECs intradermally around the full-thickness excisional skin wounds of db/db mice. We further explored related mechanisms through in vivo and in vitro experiments, mainly focusing on inflammation and neovascularization.

## 2. Materials and Methods

### 2.1. Cell Isolation and Culture

All research proposals were permitted by the Ethics Committee of Changhai Hospital, Shanghai, China. We first obtained informed consent from parturients and then got placentas directly from parturients immediately after cesarean section. All donors had no human hepatitis B and C, syphilis, and HIV verified by serological tests. Collection and isolation of hAECs was performed as described previously [9]. hAECs were cultured in a high-glucose DMEM supplemented with 10% FBS (complete medium), and hAECs at passage 2-3 were used.

Mouse macrophages were separated from bone marrow of wild-type C57BL/6 mice and cultured in a complete medium, and primary-cultured macrophages were used for further cell experiments as previously described [16]. Human umbilical vein endothelial cells (HUVECs) were purchased from ScienCell (San Diego, USA) and cultured in the endothelial cell medium (ECM, Gibco, Life Technologies) supplemented with 10% FBS, and HUVECs between passages 2 and 4 were used for further in vitro experiments. To assess the effects of the hAEC-conditioned medium (hAEC-CM) on macrophage phenotype switch in vitro, we used IFN-*γ* and TNF-*α* (20 ng/ml each, R&D Systems) to stimulate the macrophages for 24 hours with or without hAEC-CM. Complete medium without TNF-*α* and IFN-*γ* was used as control.

### 2.2. Preparation of the hAEC-Conditioned Medium (hAEC-CM)

We prepared hAEC-CM using a method analogous to the one described previously [17]. In brief, hAECs were digested with trypsin and then 2 × 10^6^ hAECs were cultured in a 100 mm plate overnight. We changed the complete culture medium into a 10 ml serum-free high-glucose DMEM and cultured for another 24 hours. The supernatant was collected and concentrated to 10 times the concentration of the collected hAEC-CM as the final hAEC-CM by using Amicon Ultra-15 ultrafiltration conical tubes (Millipore, 3 kDa).

### 2.3. Animal Model and Treatment

All animal protocols were complied with the rules of the animal use and care committee of Changhai Hospital, Shanghai, China. The db/db mouse was a widely accepted animal model of type 2 diabetes. We purchased the male and 8–12 weeks old db/db mouse (C57BL/KsJ, leptin receptor-deficient diabetes) from SLAC Laboratory Animal Co. Ltd., Shanghai, China. Only mice with a >300 mg/dl blood glucose level were included for further animal experiments. Mice were anesthetized, and then two symmetric splint wounds (10 mm diameter) with full-thickness skin defect were made on the back of each mouse based on the previous study [18]. We divided the mice equally into 2 groups: hAEC group and blank group (PBS group). 1 × 10^6^ cells suspended in 100 *μ*l PBS were injected intradermally around the wound. Photographs of wounds were taken regularly, and then, the wound healing rate was figured by Image-Pro Plus software.

### 2.4. Skin Histological Analysis and Immunohistochemistry

The wounds with the margins were excised at day 10 after wounding, fixed in 10% formalin, and then embedded in paraffin for hematoxylin and eosin (H&E) staining and immunohistochemistry staining. We measured the wound bed area of each section stained with H&E using Image-Pro Plus software. Angiogenesis in day 10 wounds was observed by immunohistochemistry using CD31 (Santa Cruz, USA) as the primary antibody and then stained with DAB (Thermo, USA). For evaluation the number of M1 and M2 macrophages in day 10 wounds, we counterstained the sections by immunofluorescence with CD68 (Abcam) and iNOS (Abcam) to calculate M1 macrophages and with CD68 and CD206 (Abcam) to calculate M2 macrophages. All sections were finally stained with DAPI.

### 2.5. ELISA

Wounds with the margins at day 10 were excised with a skin biopsy punch and homogenized in cold PBS by a Dounce homogenizer. After being sonicated, the homogenates were centrifuged at 4°C (10,000 rpm, 20 minutes). Supernatants were collected, and expression levels of IL-6, IL-1*β*, TNF-*α*, VEGF, TGF-*β*1, and IGF-1 were calculated by ELISA. All ELISA kits were purchased from R&D Systems and used according to the manufacturer's instruction.

### 2.6. HUVEC Proliferation Assay

Proliferation of HUVECs was evaluated by a CCK-8 Kit (Liankebio, China) in conformity to the instruction of the manufacturer. In brief, HUVECs were digested with trypsin, and 5 × 10^3^ cells were plated into each well of a 96-well plate (BD Falcon). After being cultured in a complete medium for 24 hours, the cells were then cultured in hAEC-CM for another 72 hours. Complete medium was treated as a positive control medium, and serum-free high-glucose DMEM was used as a negative control medium.

### 2.7. HUVEC Tube Formation Assay

We performed the tube formation assay in conformity to the instructions of the manufacturer. Matrigel (BD Biosciences, USA) was diluted with serum-free DMEM to 5 mg/ml, and 50 *μ*l gel was plated into each well of a 96-well plate. 4 × 10^4^ HUVECs suspended in 100 *μ*l hAEC-CM were plated into each well. After incubating HUVECs for 6 hours, we took photographs with a microscope system (Leica, Germany) and then calculated the capillary-like structures. Complete medium was treated as a positive control medium, and serum-free high-glucose DMEM was used as a negative control medium.

### 2.8. HUVEC Migration Assay

We performed HUVEC migration assay by a transwell chamber with 8 *μ*m pore size (BD Biosciences, USA). The upper chamber was plated with 1 × 10^5^ HUVECs suspended in serum-free DMEM, and the bottom chamber was added with 600 *μ*l hAEC-CM. After 24 hours, we fixed the filters with 10% formalin, stained them with 0.1% crystal violet (Sigma, USA), and then counted the migrated cells by Leica QWin image analysis software. Complete medium was treated as a positive control medium, and serum-free high-glucose DMEM was used as a negative control medium.

### 2.9. Western Blot Analysis

The macrophages were lysed in lysis buffer after washing with ice cold PBS buffer. Lysates were centrifuged, and the supernatants were collected for Western blot analysis. The protein concentration was determined by the BCA protein assay kit. Western blot analysis was performed with primary antibody against iNOS, CD206, and GAPDH as previously described [19].

### 2.10. Detection of Growth Factors and Inflammatory Cytokines in hAEC-CM

hAEC-CM was collected to detect the expression of growth factors and inflammatory cytokines by using human growth factor antibody array G1 and human inflammation antibody array G1 (RayBiotech Inc.), respectively, in accordance with the manufacturer's protocol.

### 2.11. Statistical Analysis

We expressed the results as mean ± SD and analyzed the data using two-tailed Student's *t*-test or one-way ANOVA by SPSS 16.0. *P* < 0.05 was considered as statistical significance.

## 3. Results

### 3.1. Topical Administration of hAECs Accelerated Diabetic Wound Healing

**Figure 1:**
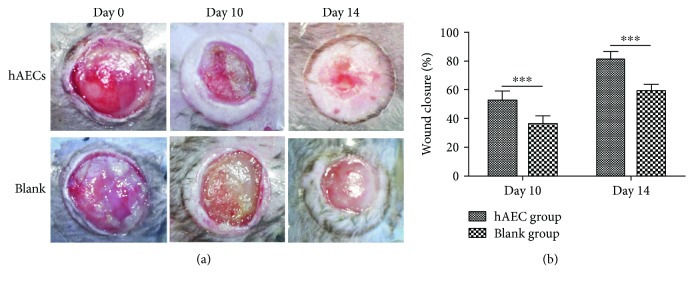
Topical administration of hAECs accelerated diabetic wound healing. (a) Diabetic wounds were treated with hAECs or PBS, examined at day 0, day 10, and day 14 after wounding, and then digitally photographed. (b) Quantitative analysis of wound closure demonstrating a higher wound healing rate in the hAEC-treated group. Data was shown as means ± SD; *n* = 6; ^∗∗∗^*P* < 0.001.

**Figure 2:**
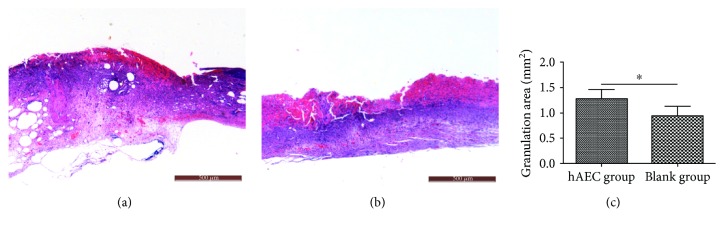
Histomorphometric analysis of diabetic wounds. Representative H&E staining of wound sections at day 10 from the hAEC group (a) and PBS group (b). Quantitative analysis of the granulation tissue area, showing the increased granulation tissue formation in the hAEC-treated group (c). Data was shown as means ± SD; *n* = 6; ^∗^*P* < 0.05; scale bar = 500 *μ*m.

We treated diabetic wounds with hAECs or PBS and then examined at day 0, day 10, and day 14 after wounding (Figure 1(a)). At day 10, the wound healing rate was significantly higher in the hAEC-treated group (52.16 ± 6.80%) than that in the PBS-treated group (35.76 ± 6.19%, *P* < 0.001, Figure 1(b)). The gap of the wound healing rate between the two groups gradually increased along with time. Furthermore, we performed H&E staining (Figures 2(a) and 2(b)) and found that the granulation tissue area was significantly higher in day 10 hAEC-treated wounds than that in PBS-treated wounds (Figure 2(c)).

### 3.2. hAECs Promoted Macrophage Phenotype Switch from M1 Macrophage to M2 Macrophage in Diabetic Wounds

**Figure 3:**
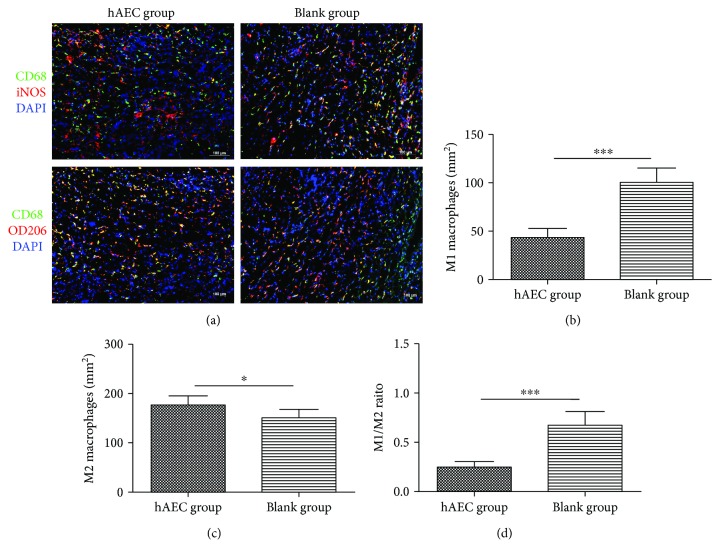
Macrophage phenotype was polarized toward M2 macrophage in the hAEC-treated group. (a) Representative immunofluorescent staining of M1 and M2 macrophages in day 10 wounds. M1 macrophages were shown in the upper panel to be yellow as CD68^+^iNOS^+^DAPI^+^ triple-positive staining, and M2 macrophages were shown in the lower panel to be yellow as CD68^+^CD206^+^DAPI^+^ triple-positive staining. (b, c, and d) The number of M1 macrophages was significantly reduced in the hAEC group (b), whereas the number of M2 macrophages was increased (c), resulting in a lower M1/M2 ratio (d). Data was shown as means ± SD; *n* = 6; ^∗^*P* < 0.05; ^∗∗∗^*P* < 0.001; scale bar = 100 *μ*m.

**Figure 4:**
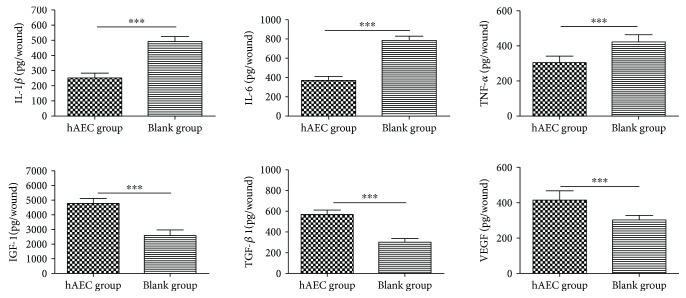
hAECs switched proinflammatory status into prohealing status of diabetic wounds. Expression levels of IL-6, IL-1*β*, TNF-*α*, VEGF, TGF-*β*1, and IGF-1 in day 10 diabetic wounds were detected by ELISA, showing reduced levels of proinflammatory cytokines and increased expression levels of prohealing cytokines in the hAEC group. Data was shown as means ± SD; *n* = 6; ^∗∗∗^*P* < 0.001.

The numbers of M1 and M2 macrophages in day 10 wounds were assessed by immunofluorescence (Figure 3(a)). Using iNOS as a marker of M1 macrophage and CD206 as a marker of M2 macrophage, we found that hAEC-treated wounds had fewer M1 macrophages (Figure 3(b)), more M2 macrophages (Figure 3(c)), and a significantly lower M1/M2 ratio (Figure 3(d)). We then measured the expression of inflammatory cytokines and prohealing cytokines in day 10 wounds using ELISA. The expression levels of proinflammatory cytokines IL-1*β*, IL-6, and TNF-*α* were significantly reduced in the hAEC group, and the levels of prohealing cytokines VEGF, TGF-*β*1, and IGF-1 were significantly increased in the hAEC group when compared with the PBS group (Figure 4), indicating that hAECs could switch proinflammatory status into prohealing status of diabetic wounds.

### 3.3. hAECs Increased Capillary Density in Diabetic Wounds

**Figure 5:**
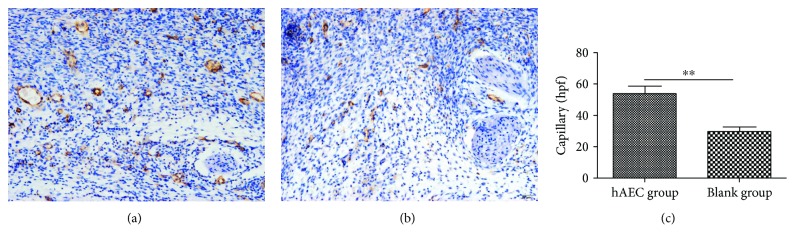
hAECs increased capillary density in diabetic wounds. Representative immunohistochemistry staining of CD31 at day 10 after wounding in the hAEC group (a) and PBS group (b). Statistical analysis of CD31^+^ newly formed vessels showed that neovascularization was more obvious in hAEC-treated wounds (c). Data was shown as means ± SD; *n* = 6; ^∗∗^*P* < 0.01; scale bars: 50 *μ*m (a, b).

CD31 was a blood vessel endothelium cell marker, and immunohistochemistry staining of CD31 demonstrated that the newly formed vessels in the hAEC group were more obvious than that in the PBS group in day 10 wounds of db/db mice (Figures 5(a) and 5(b)). Capillary density in hAEC-treated wounds was 53.8 ± 11.8/hpf (high-power field), whereas the number was 29.7 ± 7.3/hpf in PBS-treated wounds (*P* < 0.01, Figure 5(c)).

### 3.4. hAEC-CM Promoted Phenotype Switch of Macrophages toward M2-Like *In Vitro*

**Figure 6:**
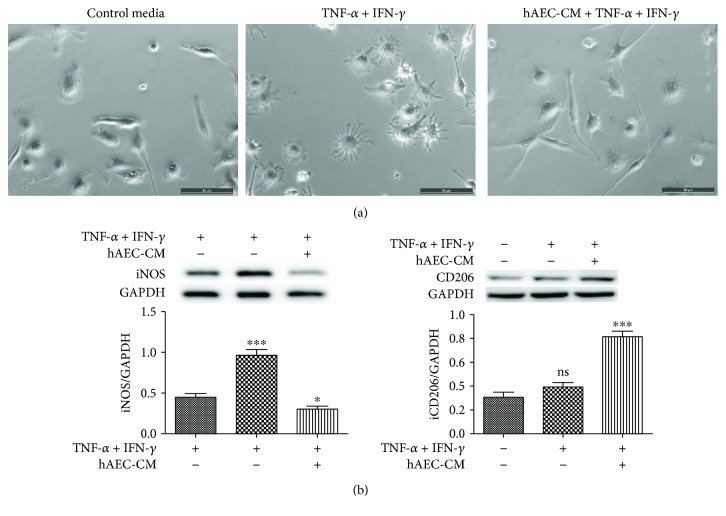
hAEC-CM promoted phenotype switch of macrophages toward M2-like in vitro. (a) Changes of macrophages in morphology when treated with IFN-*γ* and TNF-*α* with or without hAEC-CM. Complete medium without IFN-*γ* and TNF-*α* was treated as the control medium. (b) Protein expression levels of iNOS and CD206 when treated with the control medium, IFN-*γ* + TNF-*α*, or IFN-*γ* + TNF-*α* + hAEC-CM were measured by Western blot. Data was shown as means ± SD; *n* = 3; ^∗^*P* < 0.05; ^∗∗∗^*P* < 0.001; and ns: no significance; scale bars: 50 *μ*m (a).

The primary-cultured macrophages were small and round cultured in control media. When treated with IFN-*γ* + TNF-*α*, many macrophages processed multiple protrusions and showed dendritic morphology, whereas IFN-*γ* + TNF-*α* + hAEC-CM guided elongation of macrophages (Figure 6(a)). Moreover, hAEC-CM significantly increased the protein expression of CD206 and decreased the protein expression of iNOS measured by Western blot (Figure 6(b)). Taken together, these results enunciated that hAEC-CM could guide macrophage polarization toward M2-like in morphology and phenotype.

### 3.5. hAEC-CM Promoted Migration, Proliferation, and Tube Formation of HUVECs *In Vitro*

**Figure 7:**
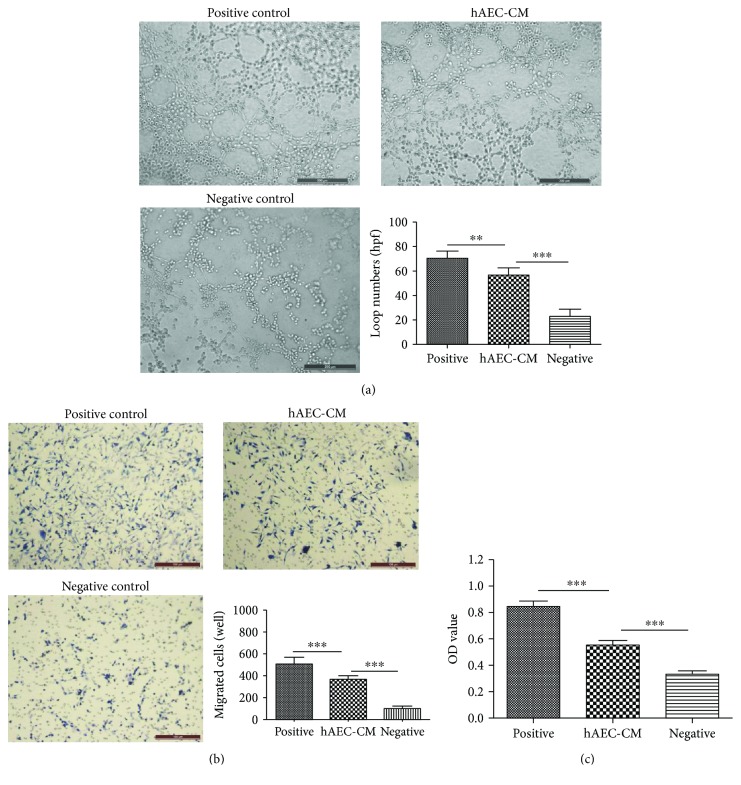
hAEC-CM exhibited chemoattractive, mitogenic, and angiogenic effects on HUVECs in vitro. (a) The tube formation assay was performed to investigate the effect of hAEC-CM on the angiogenic capacity of HUVECs. (b and c) Effect of hAEC-CM on the migration (b) and proliferation (c) of HUVECs was measured by transwell assay and CCK-8 assay, respectively. Complete medium was treated as positive control medium and serum-free DMEM as negative control medium, respectively. Data was shown as means ± SD; *n* = 6; ^∗∗^*P* < 0.01; and ^∗∗∗^*P* < 0.001. Scale bars: 200 *μ*m (a); 100 *μ*m (b).

The tube formation assay was performed to investigate the effect of hAEC-CM on the angiogenic capacity of HUVECs. We found that more networks formed when treated with hAEC-CM (Figure 7(a)). In addition, hAEC-CM significantly promoted the migration and proliferation of HUVECs performed by transwell assay and CCK-8 assay, respectively (Figures 7(b) and 7(c)).

### 3.6. Inflammatory Cytokines and Growth Factors in hAEC-CM

**Figure 8:**
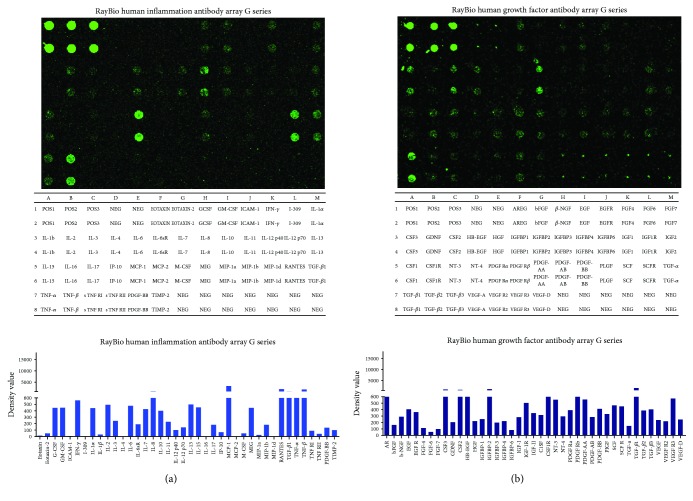
Inflammatory cytokines and growth factors in hAEC-CM. (a) RayBio human inflammatory cytokine antibody array was used to detect a panel of inflammatory cytokines in hAEC-CM. (b) RayBio human growth factor antibody array was used to detect a panel of growth factors in hAEC-CM.

Of the 40 inflammatory cytokines showed in the human inflammation antibody array, IL-8, MCP-1, RANTES, MIP-1b, TGF-*β*1, TNF, and IL-13 in hAEC-CM represented the highest expression levels (Figure 8(a)). Of the 41 growth factors represented in the human growth factor antibody array, CSF2, CSF3, HB-EGF, IGFBP-2, CSF1R, PDGF, and TGF-*β*1 in hAEC-CM demonstrated the highest expression levels (Figure 8(b)). The results showed similar kinds of inflammatory cytokines and growth factors representing the higher expression levels with the previous study, which have performed a cytokine array containing 507 human cytokines on the hAEC-derived-conditioned medium [13].

## 4. Discussion

hAECs are nontumorigenic, highly abundant, and low immunogenic and possess multipotent differentiation ability, which make them become ideal alternative stem cell source for regenerative medicine. Previous studies have demonstrated the therapeutic potential of hAECs in various tissue repairs, including lung injury [10, 11, 14], brain injury [20], myocardial infarction [21], kidney injury [22], and liver fibrosis [23, 24]. Nonetheless, the therapeutic effect of hAECs on diabetic wound healing is still unknown. In this study, we first reported that hAECs significantly accelerated the diabetic wound healing rate and granulation tissue formation, at least partially, by regulating inflammation and promoting neovascularization.

Macrophage polarization plays an important part in normal wound healing progression. During the inflammatory phase, M1 macrophages initiate an urgent inflammatory response, whereas during the proliferative phase, M2 macrophages accelerate neovascularization and granulation tissue formation [25]. A poorly healing wound such as a diabetic wound is characterized by persevering inflammatory response with protracted aggregation of M1 macrophages along with high-level expression of inflammatory factors and low-level expression of prohealing cytokines [26, 27]. Previous studies have shown that promoting macrophage phenotype switch from the M1 macrophage to the M2 macrophage could accelerate diabetic wound healing effectively [28–30]. hAECs could reduce fibrosis and promote repair of damaged tissue by modulating inflammation and promoting macrophage polarization from M1 macrophage to M2 macrophage [14, 31], but the effect of hAECs on macrophage phenotype switch in diabetic wounds is at present unknown. In this study, we found that hAECs could promote macrophage phenotype switch from M1 macrophage to M2 macrophage and switch proinflammatory status into prohealing status of diabetic wounds. These results from our in vitro and in vivo experiments are consistent with the previously reported data. Using RayBio human inflammation antibody arrays, we found that hAECs secreted IL-13 and TGF-*β*1, which contributed to the polarization of macrophages [32, 33]. This finding may partly explain the promoting effect of hAECs on macrophage polarization in vitro and in vivo.

Another factor contributing to refractory diabetic wound healing is the inhibited neovascularization [27]. Previous studies have reported that hAECs could promote neovascularization in the damaged tissues, including chemotherapy-induced ovarian damage and hyperoxia-induced lung injury [13, 34]. However, whether hAECs could promote neovascularization in diabetic wounds is currently unaware. In this study, we found increased capillary density in hAEC-treated wounds. Two likely underlying reasons may lead to this result. First, we suspected that the prohealing local environment of the hAEC-treated diabetic wounds associated with increased number of M2 macrophages and elevated levels of various growth factors promoted neovascularization indirectly. Second, our results showed that hAEC-CM could promote proliferation, migration, and tube formation of endothelial cells, suggesting the paracrine effects of hAECs on neovascularization directly. Using RayBio human inflammation antibody arrays and growth factor antibody arrays, we found that a variety of active peptides (PDGF, IL-8, and TGF-*β*1) were secreted from hAECs and these peptides are strongly associated with neovascularization [35–37]. This finding may partly explain the direct promoting effect of hAECs on neovascularization.

The present study has its limitation. First, notwithstanding some potential active peptides screened by RayBio antibody arrays may explain the effects of hAECs on the function of macrophages and endothelial cells; further studies are still necessary to find out the key active peptides and illuminate related signaling passway. Second, although the potential mechanisms of hAECs in promoting tissue repairs are not yet well-understood, the main therapeutic mechanisms of stem cells have been explained by paracrine effects rather than transdifferentiation, considering that only a fraction of grafted hAECs could survive in the damaged tissues [13, 20, 38]. Furthermore, impaired diabetic wound healing is characterized by hypoxia, impaired neovascularization, and excessive inflammation, which are extremely detrimental to the survival of hAECs in the wounds. For all the above reasons, it suggests that hAECs are hard to survive in the diabetic wounds for a long period of time and paracrine effects rather than transdifferentiation may play a key role of hAECs in promoting diabetic wound healing. However, further researches are still necessary to provide the direct evidences to determine the fate of hAECs after transplantation onto diabetic wounds.

## 5. Conclusions

Taken together, the results of the present study first showed that hAECs significantly accelerated the diabetic wound healing rate and granulation tissue formation, at least partially, by regulating inflammation and promoting neovascularization. hAECs are nontumorigenic, highly abundant, and low immunogenic, suggesting that it might be a safe and effective therapeutic approach to treat diabetic ulcers and other chronic wounds in clinics.

## Data Availability

The data used to support the findings of this study are available from the corresponding author upon request.
